# Supplementary material for: The transcription factor EHF promotes the maturation and immunosuppression of conventional dendritic cells
Source: Nat Commun. 2026 Feb 23;17:3094. doi: 10.1038/s41467-026-69959-z (PMC13039115; doi:10.1038/s41467-026-69959-z)
Supplement: Supplementary file 1 — Supplementary information [file 41467_2026_69959_MOESM1_ESM.pdf]

The transcription factor EHF promotes the maturation and immunosuppression of conventional dendritic cells

This Supplementary Information contains Supplementary Figures 1-6 that corresponds to the main figures 1-6, gating strategies, a graphical abstract and Supplementary Tables 1-3.

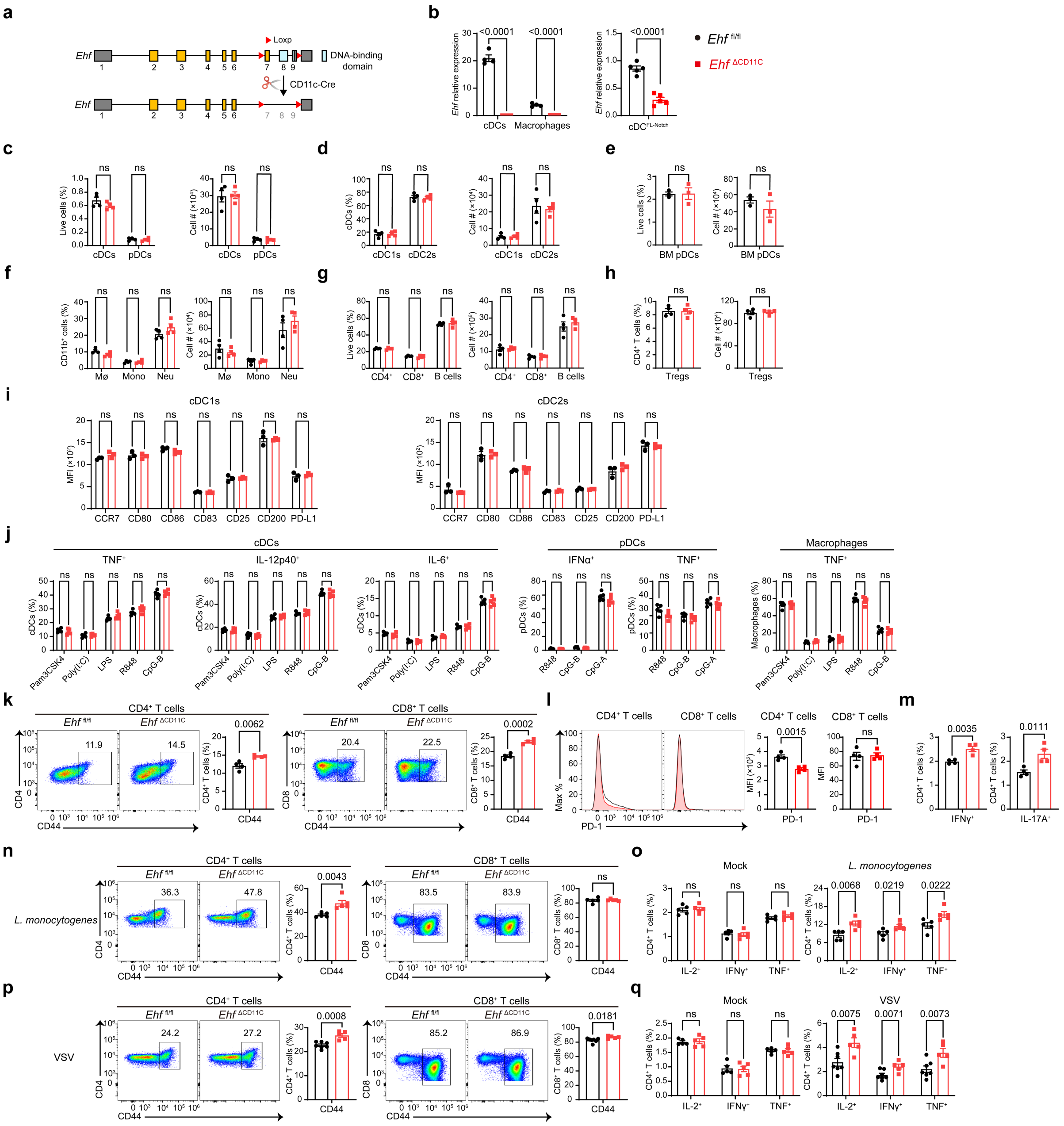

Supplementary Figure 1 : (a) Schematic of the *Ehf* conditional knockout genetic strategy. **(b)** Relative *Ehf* expression in FACS-sorted splenic cDCs, macrophages and $cDC^{FL\text{-}Notch}$ from *Ehf* $^{fl/fl}$ and *Ehf* $^{\Delta CD11C}$ mice was measured via qPCR (cDCs and macrophages, n=4 mice per group; $cDC^{FL\text{-}Notch}$, n=5 mice per group). **(c)** The proportions of splenic cDCs and pDCs and their cell numbers in *Ehf* $^{fl/fl}$ and *Ehf* $^{\Delta CD11C}$ mice were measured via FACS (n=4 mice per group). **(d)** The proportions of splenic cDC1s and cDC2s and their cell numbers in *Ehf* $^{fl/fl}$ and *Ehf* $^{\Delta CD11C}$ mice were measured via FACS (n=4 mice per group). **(e)** The proportions of BM pDCs and their cell numbers in *Ehf* $^{fl/fl}$ and *Ehf* $^{\Delta CD11C}$ mice were measured via FACS (n=3 mice per group). **(f)** The proportions of splenic $CD11b^+$ myeloid cells and their cell numbers in *Ehf* $^{fl/fl}$ and *Ehf* $^{\Delta CD11C}$ mice were measured via FACS (n=4 mice per group). **(g)** The proportions of splenic B cells, $CD4^+$ T cells, $CD8^+$ T cells and their cell numbers in *Ehf* $^{fl/fl}$ and *Ehf* $^{\Delta CD11C}$ mice were measured via FACS (n=4 mice per group). **(h)** The proportions of splenic

Treg cells and their cell numbers in *Ehf*$^{fl/fl}$ and *Ehf*$^{\Delta CD11C}$ mice were measured via FACS (n=4 mice per group). **(i)** Expression of the indicated surface molecules on splenic cDC1s (left) and cDC2s (right) from *Ehf*$^{fl/fl}$ and *Ehf*$^{\Delta CD11C}$ mice at steady-state was measured by MFI (n=3 mice per group). **(j)** Indicated cytokine production by FACS-sorted cDCs (spleen), pDCs (BM) and macrophages (peritoneal lavage fluid) was measured by intracellular staining in vitro after stimulation with indicated ligands for 9-12 hrs (n=5 mice per group). **(k)** The proportions of mesenteric lymph nodes $CD44^+$ T cells was measured via FACS on Day 7 after DSS treatment (n=4 mice per group). **(l)** PD-1 expression on mesenteric lymph nodes T cells was measured via FACS on Day 7 after DSS treatment (n=4 mice per group). **(m)** IFNγ and IL-17A production by mesenteric lymph nodes $CD44^+CD4^+$ T cells was measured by FACS on Day 7 after DSS treatment (n=4 mice per group). **(n)** The proportions of splenic $CD44^+$ T cells was measured via FACS on Day 5 after *L. monocytogenes* infection (n=5 mice per group). **(o)** IL-2, IFNγ and TNF production by splenic $CD44^+CD4^+$ T cells was measured by FACS on Day 5 after mock or *L. monocytogenes* infection (n=5 mice per group). **(p)** The proportions of splenic $CD44^+$ T cells was measured via FACS on Day 5 after VSV infection (*Ehf*$^{fl/fl}$, n=7 mice; *Ehf*$^{\Delta CD11C}$: n=5 mice). **(q)** IL-2, IFNγ and TNF production by splenic $CD44^+CD4^+$ T cells was measured by FACS on Day 5 after mock or VSV infection (mock, n=5 mice per group; VSV: *Ehf*$^{fl/fl}$, n=7 mice; *Ehf*$^{\Delta CD11C}$, n=5 mice). P values were calculated by two-tailed Student's t test (ns, P>0.05; *, P < 0.05; **, P < 0.01; ***, P < 0.001; ****; P < 0.0001). All the data are presented as the means ± SEMs and are representative of three independent experiments.

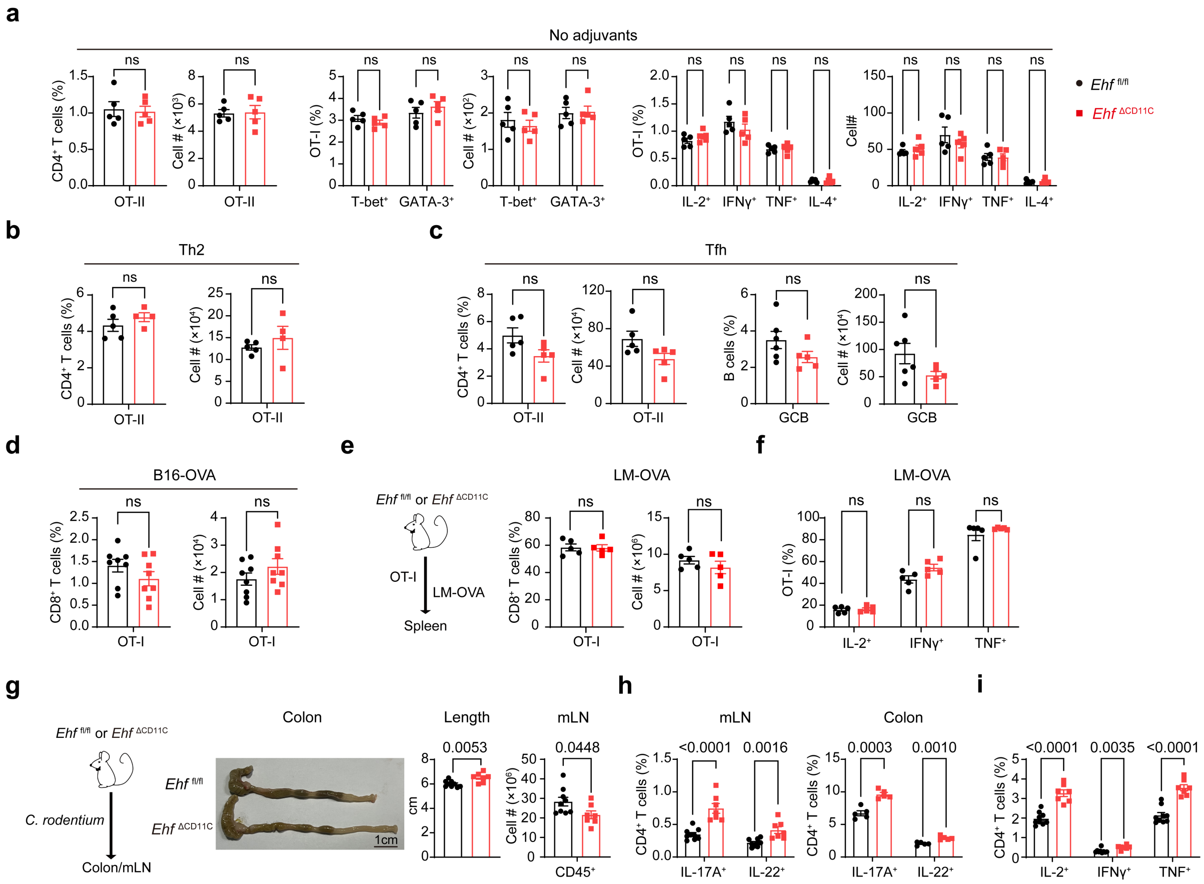

Supplementary Figure 2: (a) CD45.1$^+$ OT-II expansion, indicated cytokines or transcription factors expression by OT-II cells in popliteal lymph nodes was measured by FACS on Day 6 after i.p. injection of 1×10^6 naive OT-II cells without any adjvants (n=5 mice per group). **(b)** CD45.1$^+$ OT-II expansion in popliteal lymph nodes was measured by FACS on Day 5 after s.c. injection of papain/IFA/OVA (*Ehf* $^{fl/fl}$, n=5 mice; *Ehf* $^{\Delta CD11C}$, n=4 mice). **(c)** CD45.1$^+$ OT-II expansion and germinal center B cells (GCB) were measured by FACS on Day 5 after i.p. injection of SRBC-OVA (OT-II, n=5 mice per group; GCB: *Ehf* $^{fl/fl}$, n=6 mice; *Ehf* $^{\Delta CD11C}$, n=5 mice). **(d)** On Day 11 after B16-OVA transplantation, OT-I cells were i.v. injected. CD45.1$^+$ OT-I cell expansion in tumors was measured via FACS after 3 days (n=8 mice per group). **(e)** CD45.1$^+$ OT-I cell expansion in spleens from *Ehf*$^{fl/fl}$ and *Ehf*$^{\Delta CD11C}$ mice was measured by FACS on Day 5 after infection with LM-OVA (n=5 mice per group). **(f)** IL-2, IFNγ and TNF production by splenic OT-I cells from *Ehf*$^{fl/fl}$ and *Ehf*$^{\Delta CD11C}$ mice was measured by FACS on Day 5 after LM-OVA infection (n=5 mice per group). **(g)** Representative images of the colons (left) from *Ehf*$^{fl/fl}$ and *Ehf*$^{\Delta CD11C}$ mice, their lengths (middle), and CD45$^+$ immune cells in mesenteric lymph nodes (right) 11 days after *C. rodentium* infection are shown (*Ehf* $^{fl/fl}$, n=9 mice; *Ehf* $^{\Delta CD11C}$, n=7 mice; scale bar=1 cm). **(h)** IL-17A and IL-22 production by CD44$^+$CD4$^+$ T cells from mesenteric lymph nodes or colons was measured by FACS on Day 11 post *C. rodentium* infection (mLN: *Ehf* $^{fl/fl}$, n=9 mice; *Ehf* $^{\Delta CD11C}$, n=7 mice; colon: n=5 mice per group). **(i)** IL-2, IFNγ and TNF production by CD44$^+$CD4$^+$ T cells of mesenteric lymph nodes was measured by FACS on Day 11 post *C. rodentium* infection (*Ehf* $^{fl/fl}$, n=9 mice; *Ehf* $^{\Delta CD11C}$, n=7 mice). P values were calculated by two-

tailed Student’s t test (ns, $P>0.05$; *, $P < 0.05$; **, $P < 0.01$; ***, $P < 0.001$; ****; $P < 0.0001$). All the data are presented as the means ± SEMs and are representative of three independent experiments.

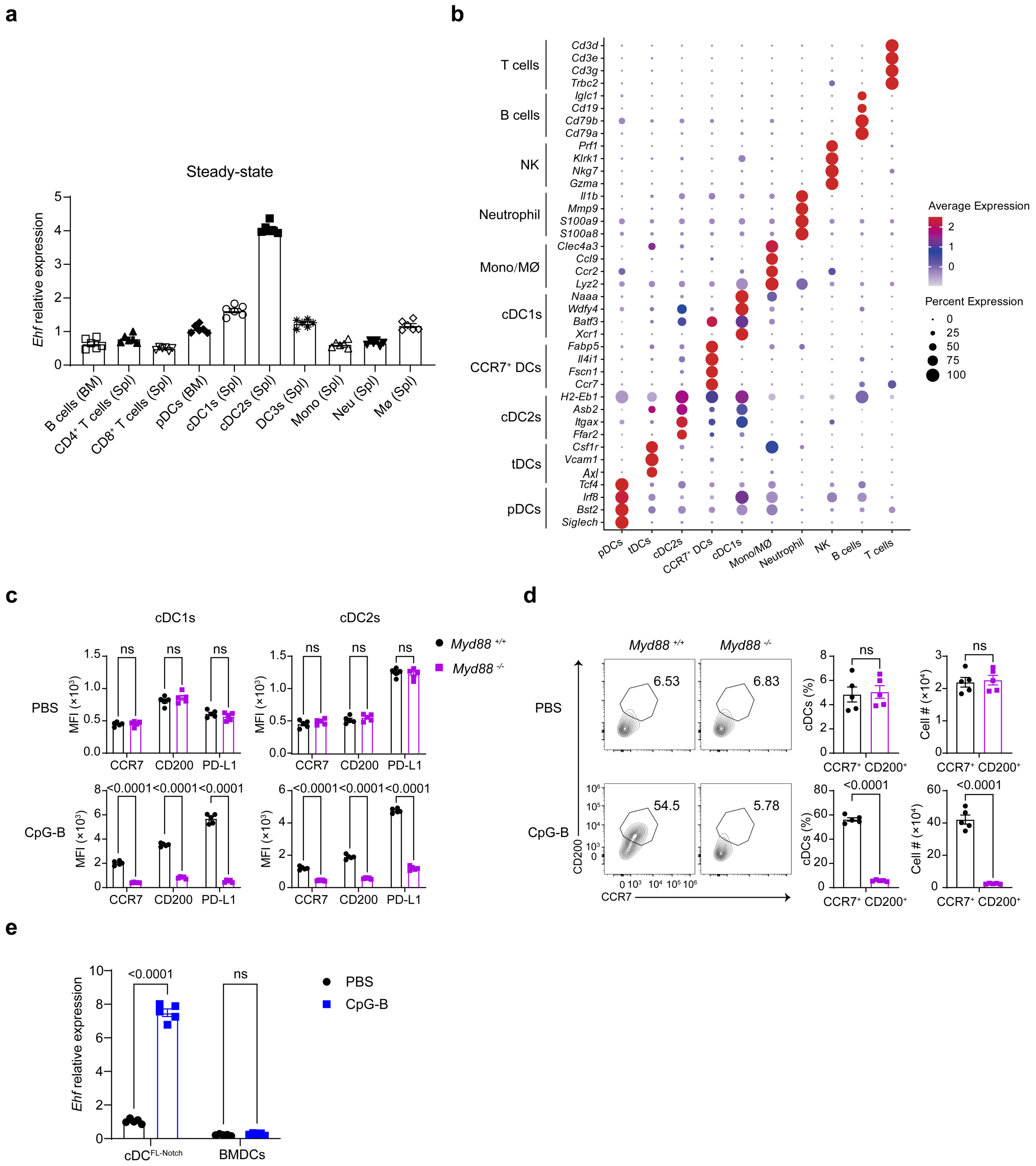

Supplementary Figure 3: (a) *Ehf* transcript levels were measured via qPCR in the indicated FACS-sorted immune populations from steady-state B6 mice (n=6 independent runs with 10 pooled B6 mice). **(b)** Dot plot showing differentially expressed marker genes cataloged in each cluster from the murine scRNA-seq data shown in **Fig. 3b**. **(c)** Expression of the indicated surface molecules on splenic cDC1s and cDC2s from *Myd88* $^{+/+}$ and *Myd88* $^{-/-}$ mice was measured by FACS at 12 hrs after i.p. injection of PBS or CpG-B (n=5 mice per group). **(d)** The proportions of splenic CCR7^{+}CD200^{+} cDCs in *Myd88* $^{+/+}$ and *Myd88* $^{-/-}$ mice and their cell numbers were measured via FACS at 12 hrs after i.p. injection of PBS or CpG-B (n=5 mice per group). **(e)** GM-CSF-derived BMDCs or cDC$^{FL\text{-}Notch}$ were cultured from B6 bone marrow, and *Ehf* transcript levels were measured via qPCR after stimulated with PBS or CpG-B for 14 hrs (n=5 mice per group). P values were calculated by two-tailed Student's t test (ns, P>0.05; *, P < 0.05; **, P < 0.01; ***, P < 0.001; ****; P < 0.0001). All the data are

presented as the means ± SEMs and are representative of three independent experiments, except for **b**, which was from one experiment.

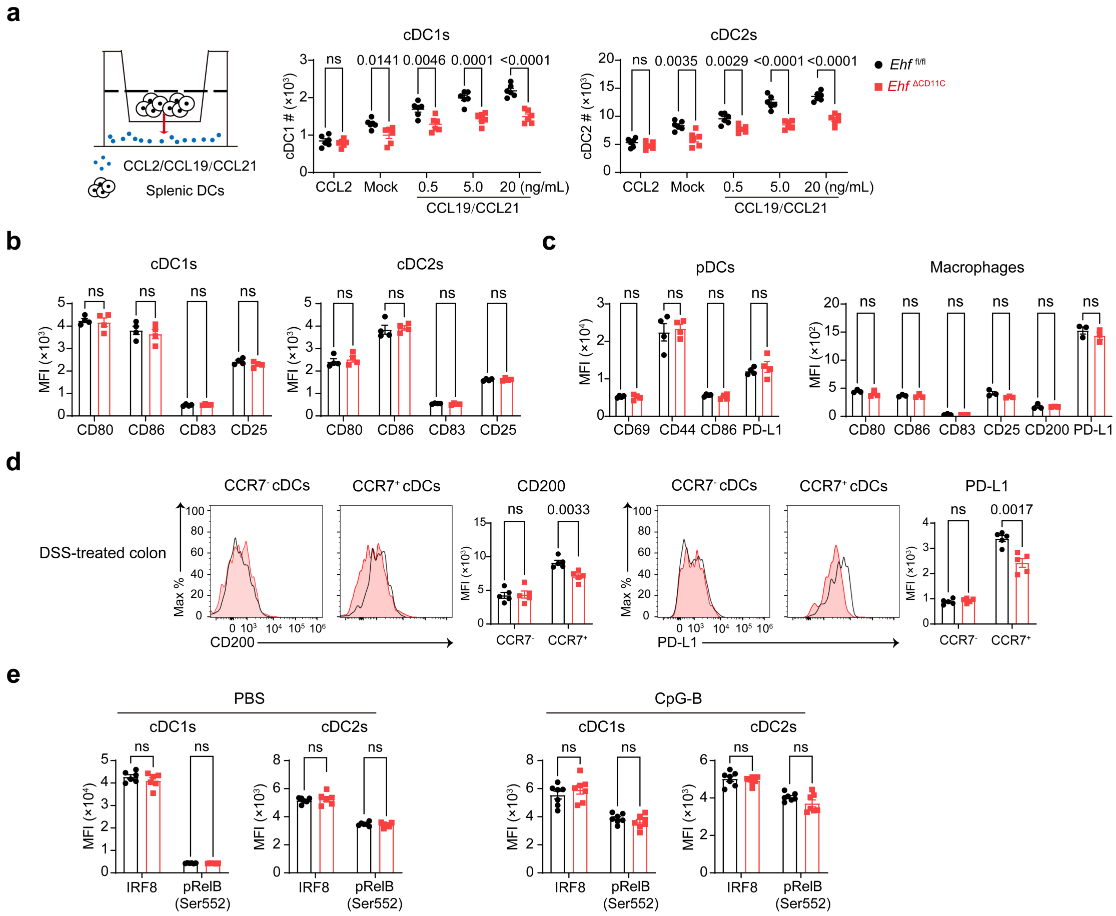

Supplementary Figure 4: (a) Purified cDCs from *Ehf* $^{fl/fl}$ and *Ehf* $^{\Delta CD11C}$ mice were seeded in the top chambers with 20 ng/mL CCL2 or indicated concentrations of CCL19 and CCL21 at the bottom of transwell. After 12 hrs, the numbers of cDC1s or cDC2s migrated to the bottom of transwell were counted (n=6 mice per group). **(b)** Expression of the indicated surface molecules on splenic cDC1s (left) and cDC2s (right) from *Ehf* $^{fl/fl}$ and *Ehf* $^{\Delta CD11C}$ mice was measured by FACS at 12 hrs after i.p. injection of CpG-B (n=4 mice per group). **(c)** Expression of the indicated surface molecules on BM pDCs (left) and splenic macrophages (right) from *Ehf* $^{fl/fl}$ and *Ehf* $^{\Delta CD11C}$ mice was measured by FACS after CpG-A (pDCs, in vitro) or CpG-B (macrophages, in vivo) stimulation for 9-12 hrs (pDCs, n=4 mice per group; macrophages, n=3 mice per group). **(d)** CD200 and PD-L1 expression on colon CCR7$^-$ cDCs or CCR7$^+$ cDCs was measured via FACS on Day 7 after DSS treatment (n=5 mice per group). **(e)** IRF8 or pRelB (Ser552) expression in splenic cDC1s and cDC2s from *Ehf* $^{fl/fl}$ and *Ehf* $^{\Delta CD11C}$ mice was measured via intracellular staining at 12 hrs after i.p. injection of CpG-B (PBS, n=6 mice per group; CpG-B, n=7 mice per group). P values were calculated by two-tailed Student's t test (ns, P>0.05; *, P < 0.05; **, P < 0.01; ***, P < 0.001; ****; P < 0.0001). All the data are presented as the means ± SEMs and are representative of three independent experiments.

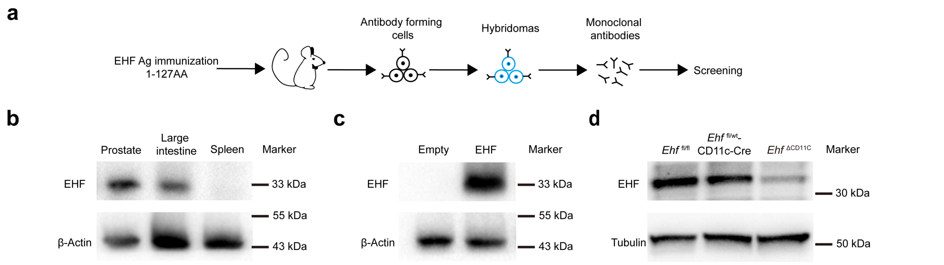

e

Description	Count	Gene ID
Leukocyte migration	43	*Tnfsf4/F11r/Slamf9/Abr/Ccl2/Ccl5/Ccl3/Ccl4/Ccr7/Pecam1/Cd300a/Edn1/Lrch1/Ext1/Rac2/Pdgfb/Cd47/Dusp1/Tnf/Vav1/Cd74/Gcnt1/Ptprj/Il1b/Sirpa/Mmp9/Lyn/Pde4b/Cdc42/Kit/Hrh1/Rps19/Rhog/Mapk3/Itgam/Myo9b/Smpd3/St3gal4/Cxcr5/Cadm1/Itga9/Cx3cr1/Ccr1*
Mononuclear differentiation	45	*Ptprc/Mr1/Tnfsf4/Ly9/Slamf9/Itpkb/Tnfaip3/Lilrb4a/Zbtb7a/Polm/Il12b/Csf2/Fnip1/Rara/Ccr7/Stat3/Batf/Cd83/Il3ra/St3gal1/Nfam1/Socs1/Plcl2/Vav1/Sos1/Egr1/Cd74/Malt1/Ptprj/Cd44/Il1b/Tpd52/Lyn/Tox/Chd7/Zc3h12a/Runx3/Rbpj/Kit/Lfng/Foxp1/Relb/Bcl3/Pou2f2/Il4i1*
Bacterial response	39	*Ly96/Slc11a1/Ncf2/Tnfsf4/Fcgr4/Tnfaip3/Lilrb4a/Sbno2/Cactin/Mir146/Il12b/Tnip1/Igtp/Cd68/Abr/Ccl2/Ccl5/Mir142/Rara/Ccr7/Nfkbia/Mtdh/Cyrib/Tnf/Malt1/Il1b/Sirpa/Lyn/Pde4b/Zc3h12a/Cd36/Tlr1/Tlr6/Scarb1/Foxp1/Irak2/Zfp36/Mapk3/Cx3cr1*
Immune activation	34	*Slc11a1/Ptprc/Tnfsf4/Cd244a/Ly9/Tnfaip3/Lilrb4a/Icosl/Sbno2/Il12b/Abr/Supt6/Rara/Ccr7/Stat3/Cd300a/Batf/Lcp1/St3gal1/Rac2/Plcl2/Cd74/Malt1/Lyn/Zc3h12a/Clnk/Kit/Lfng/Foxp1/Relb/Bcl3/Itgam/Trex1/Cx3cr1*
Immune inhibition	40	*Ptprc/Tnfsf4/Tnfaip3/Lilrb4a/Adora2a/Cactin/Il12b/Cd68/Abr/Ccl2/Rara/Cd300a/Lrch1/Tarbp2/Socs1/Cd47/Cblb/Samsn1/Dusp1/Tnf/Plcl2/Cd74/Ptprj/Dgkz/Cd44/Lyn/Zc3h12a/Smpdl3b/Runx3/Clnk/Oas3/Rps19/Il4i1/Htra1/Ubash3b/Nlrx1/Tcta/Trex1/Cx3cr1/Ccr1*
Inflammatory response	34	*Tnfsf4/Esr1/Tnfaip3/Cdk19/Adora2a/Sbno2/Il12b/Tnip1/Abr/Ccl5/Ccl3/Nfe2l1/Ccr7/Nfkbia/Pbk/Mefv/Cd47/Tnf/Fem1a/C3/Pik3ap1/Cd44/Il1b/Spata2/Lyn/Smpdl3b/Tlr6/Foxp1/Gpr4/Rps19/Zfp36/Nlrx1/Trex1/Cx3cr1*
T-cell priming	32	*Ptprc/Tnfsf4/Cd244a/Itpkb/Lilrb4a/Adora2a/Icosl/Il12b/Ccl2/Ccl5/Rara/Ccr7/Cd300a/Cd83/Cyrib/Rac2/Socs1/Cd47/Cblb/H2-Ea/Sos1/Cd74/Malt1/Cd44/Il1b/Sirpa/Tox/Sit1/Zc3h12a/Runx3/Il4i1/Trex1*
PRR signaling	22	*Ly96/Ptgs2os/Esr1/Tnfaip3/Cactin/Tnip1/Igtp/Cd300a/Nfkbia/Rftn1/Unc93b1/Slc15a3/Pik3ap1/Lyn/Smpdl3b/Cd36/Tlr1/Tlr6/Oas3/Irak2/Cyba/Nlrx1*
Transcriptional factor activity	32	*Sp100/Ptma/Tnfsf4/Aim2/Esr1/Tnfaip3/Zbtb7a/Cactin/Stat3/Psma6/Nfkbia/Edn1/Mtdh/Nfam1/Pim1/Tnf/Camk2a/Malt1/Tcf7l2/Traf1/Il1b/Hck/Crtc2/Zc3h12a/Cd36/Tlr6/Kit/Irak2/Bcl3/Slco3a1/Mapk3/Cx3cr1*
Antigen presentation	15	*Slc11a1/Mr1/Cd68/Ccr7/Psme2/Ext1/H2-Ea/Bag6/H2-D1/Rftn1/Cd74/Unc93b1/Clec4a1/Relb/Trex1*

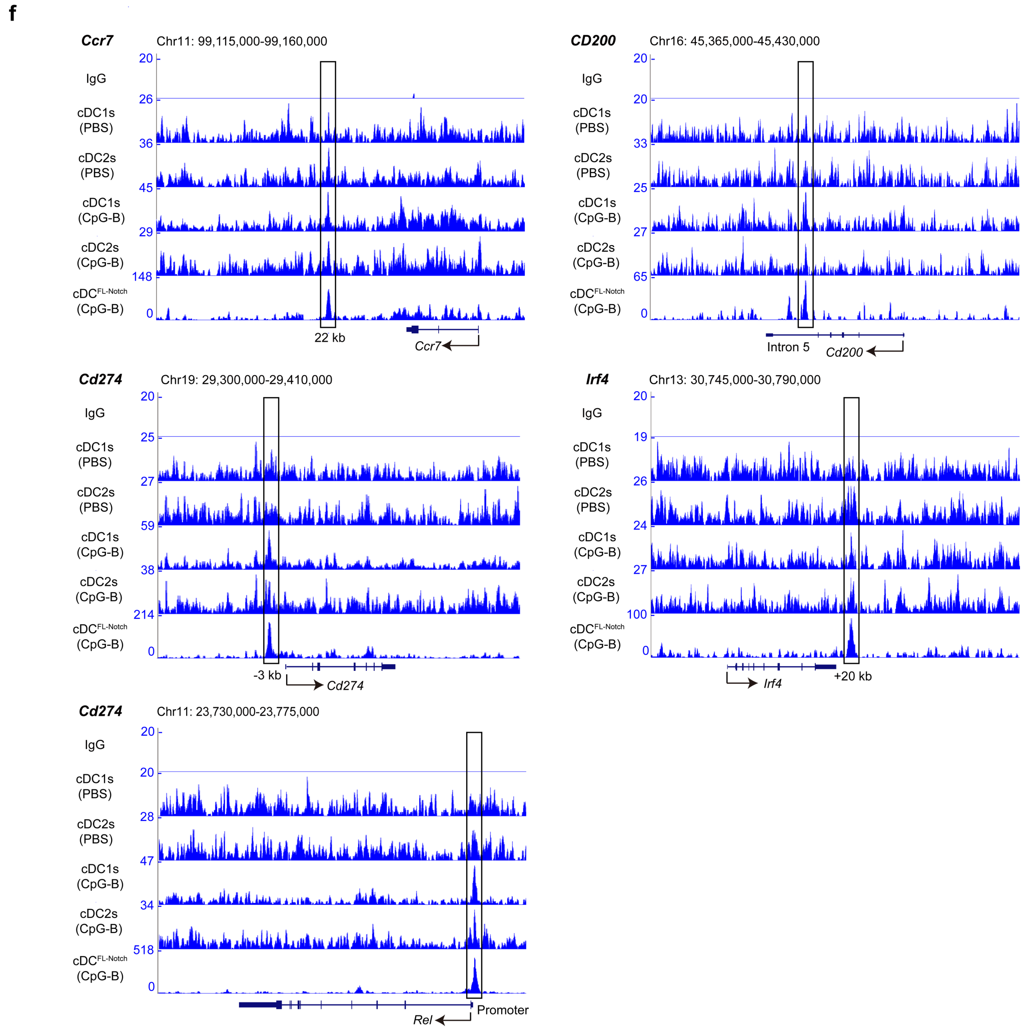

Supplementary Figure 5: (a) A schematic of the in-house generation of the anti-EHF

monoclonal antibody. **(b)** Representative Western blots of different organs from B6 mice incubated with the anti-EHF clone XL-32. **(c)** Representative Western blots of 293T cells transduced with EHF overexpression vectors or empty vectors were incubated with the anti-EHF clone XL-32. **(d)** Representative Western blots of $cDC^{FL\text{-}Notch}$ from $\textit{Ehf}^{\,fl/fl}$, $\textit{Ehf}^{\,fl/wt}$ or $\textit{Ehf}^{\,\Delta CD11C}$ mice incubated with the anti-EHF clone XL-32. **(e)** GO table of genes that corresponds to **Fig. 5d**. **(f)** CUT&TAG profiling of the DNA-binding sites by EHF around *Ccr7*, *Cd200*, *Cd274, Irf4* and *Rel* loci in splenic FACS-sorted cDC1s and cDC2s from CpG-B-treated WT mice was visualized with the UCSC genome browser. All the data are representative of two independent experiments.

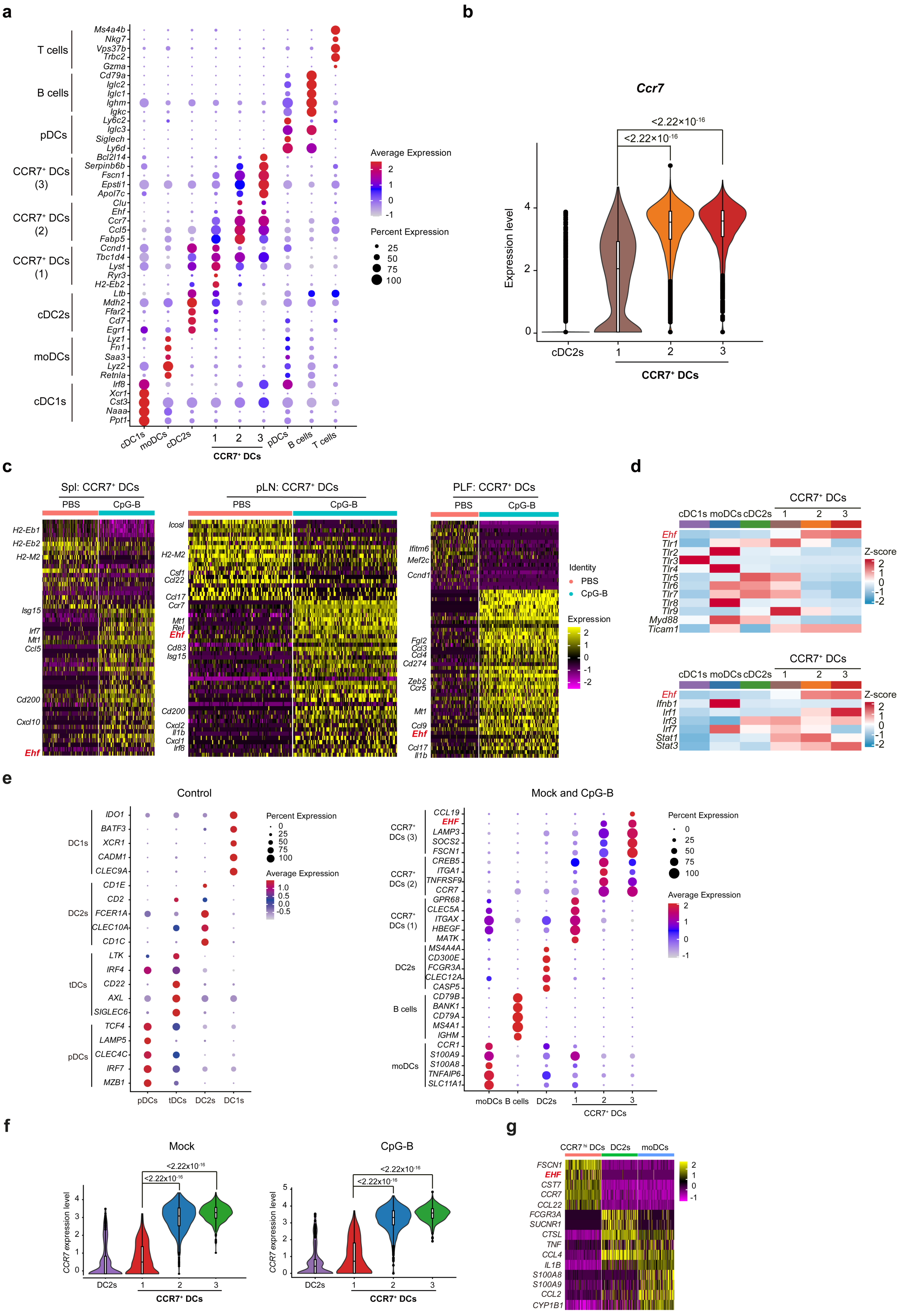

Supplementary Figure 6: (a) Dot plot showing differentially expressed marker genes cataloged in each cluster from the murine scRNA-seq data shown in **Fig. 6a-d**. **(b)** Violin plots depicting *Ccr7* expression in the indicated clusters. **(c)** Heatmaps depicting top marker genes from indicated clusters from different organs are shown. **(d)**

Heatmap depicting the average Z score of selected differentially expressed genes is shown with genes belonging to the CCR7^{hi} DC signature set as the standard. **(e)** Dot plots showing differentially expressed marker genes catalogued in clusters identified by the human scRNA-seq data shown in **Fig. 6e-f**. **(f)** Violin plots depicting *CCR7* expression in the indicated sample is shown. **(g)** Heatmap depicting the top marker genes from indicated clusters in CCR7^{hi} DCs (Cluster 3) is shown. P values were calculated by the two-sided Wilcoxon rank sum test. All the data are from one independent experiment.

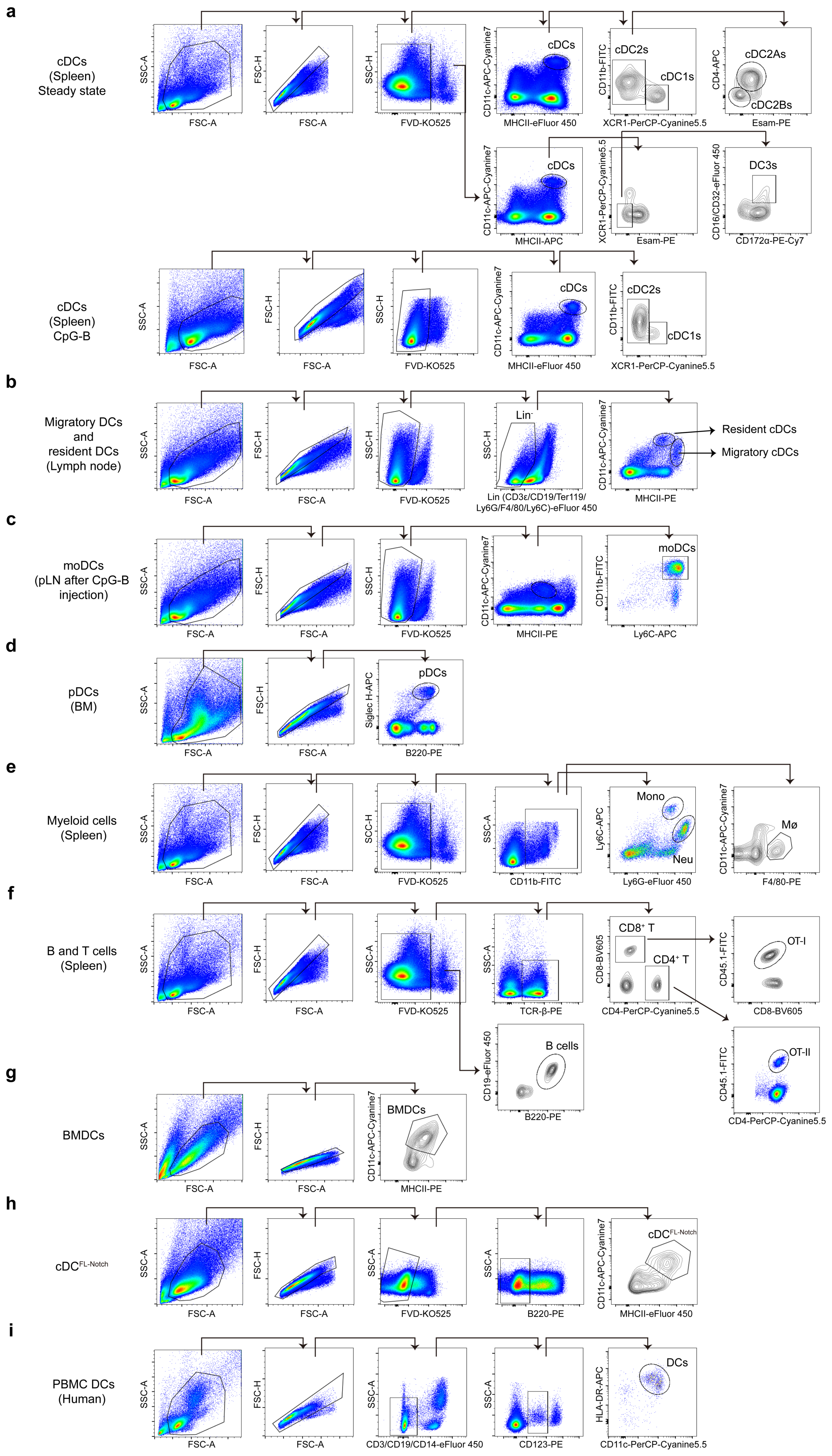

Gating strategies: The FACS gating of cDC1s, cDC2s, cDC2As, cDC2Bs and DC3s

(**a**), migratory and resident cDCs (**b**), moDCs (**c**), pDCs (**d**), monocytes, neutrophils and macrophages (**e**), B cells, $CD4^+$ T cells, $CD8^+$ T cells (**f**), BMDCs (**g**), $cDC^{FL\text{-}Notch}$ (**h**), and human DCs (**i**) are shown.

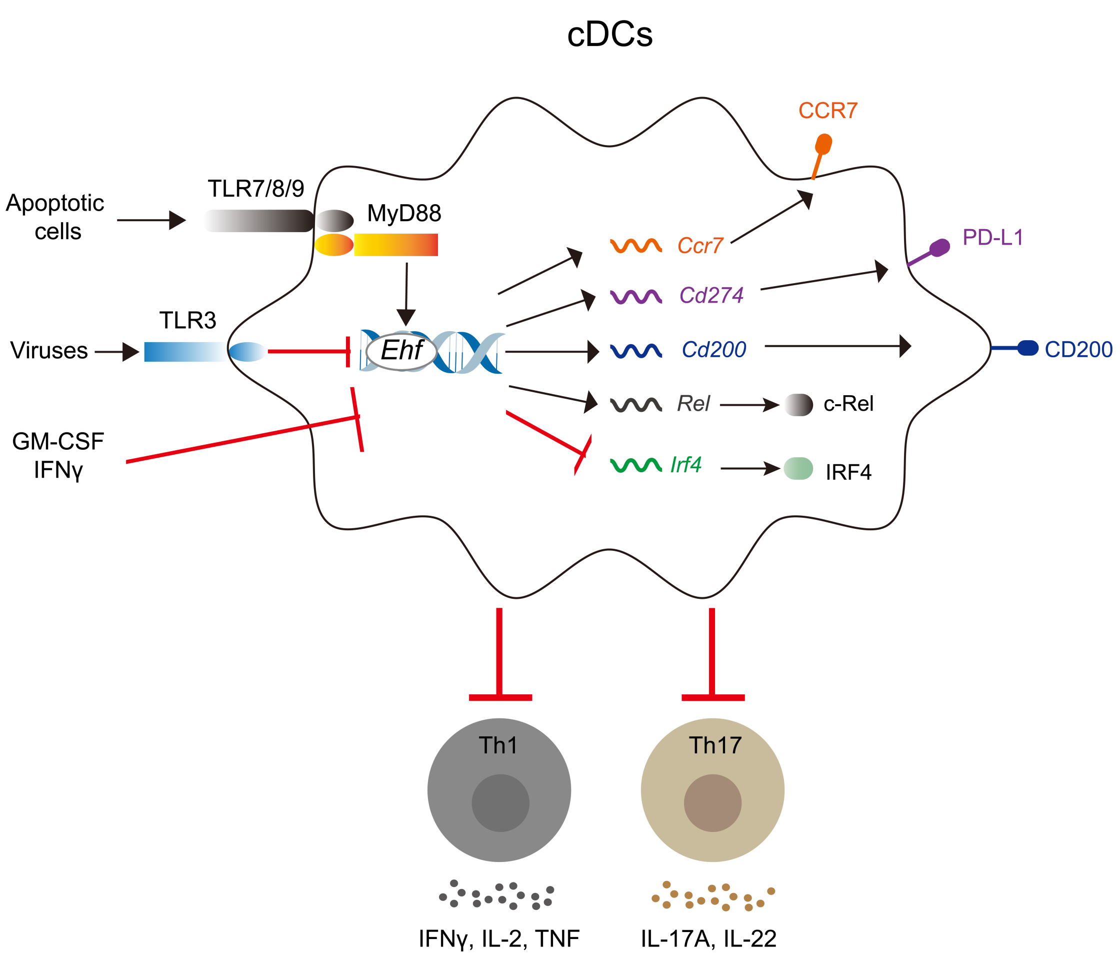

Graphic abstract

Supplementary Table 1: Antibody information

Target	Fluorophore	Clone	Source	Catalog#	Dilution
CD19	eFluor450	1D3	Invitrogen	48-0193-82	1/400
CD3ε	APC	17A2	Invitrogen	17-0032-82	1/400
CD3ε	eFluor450	500A2	Invitrogen	48-0033-82	1/400
F4/80	eFluor450	BM8	Invitrogen	48-4801-82	1/400
TER-119	eFluor450	TER-119	Invitrogen	48-5921-82	1/400
CD11b	PerCP-eFluor710	M1/70	Invitrogen	45-0112-82	1/400
CD11b	FITC	M1/70	Invitrogen	11-0112-82	1/400
B220	FITC	RA3-6B2	Invitrogen	11-0452-82	1/400
CD62L	PE-Cyanine7	MEL-14	Invitrogen	25-0621-82	1/400
BST-2	FITC	eBio927	Invitrogen	11-3172-82	1/400
SiglecH	APC	eBio440c	Invitrogen	17-0333-82	1/400
TNF	PE-Cyanine7	MP6-XT22	Invitrogen	25-7321-82	1/400
Foxp3	PE	NRRF-30	Invitrogen	12-4771-82	1/400
CD44	APC-Cyanine7	IM7	BioLegend	103028	1/400
CD44	APC	IM7	BioLegend	103012	1/400
B220	PE	RA3-6B2	BioLegend	103208	1/400
IA/IE	APC-Cyanine7	M5/114.15.2	BioLegend	107628	1/400
IA/IE	PE	M5/114.15.2	BioLegend	107608	1/400
CD4	PerCP-Cyanine5.5	GK1.5	BioLegend	100434	1/400
CD8	BV605	53-6.7	BioLegend	100743	1/400
CD25	FITC	PC61.5	Invitrogen	11-0251-82	1/400
XCR1	PerCP/Cyanine5.5	ZET	BioLegend	148208	1/400
Ly6C	FITC	HK1.4	BioLegend	128006	1/400
Ly6C	APC	HK1.4	Invitrogen	17-5932-82	1/400
F4/80	PE	BM8	BioLegend	123110	1/400
CD11c	APC-Cyanine7	N418	BioLegend	117352	1/400
CD45	APC	30-F11	BioLegend	103112	1/400
IL12p40	APC	IM7	BioLegend	505206	1/400
PD-L1	PE-Cy7	MIH5	Invitrogen	25-5982-82	1/400
PD-1	APC-Cyanine7	J43	Invitrogen	47-9985-82	1/400
CD83	PE	Michel-17	Invitrogen	12-0831-82	1/400
CD80	PE	16-10A1	Invitrogen	12-0801-82	1/400
CD86	APC	GL1	Invitrogen	17-0862-82	1/400
CD200	APC	OX-90	BioLegend	123810	1/400
CD25	FITC	PC61.5	Invitrogen	11-0251-82	1/400
CD95(Fas)	PE	15A7	eBioscience	12-0951-83	1/400
GL-7	eFluor450	GL-7	Invitrogen	48-5902-82	1/400
Ly6G/Ly6C	eFluor450	RB6-8C5	Invitrogen	48-5931-82	1/400
CD16/CD32	eFluor450	93	Invitrogen	48-0161-82	1/400
IL-4	APC	11B11	Invitrogen	17-7041-82	1/400

IL-2	eFluor450	JES6-5H4	Invitrogen	48-7021-82	1/400
CXCR5	Biotin	2G8 (RUO)	BD	551960	0.5 μg
IL-6	eFluor450	MP5-20F3	Invitrogen	48-7061-82	1/400
IRF4	eFluor660	3E4	Invitrogen	50-9858-82	0.125 μg
IRF8	PE	V3GYWCH	Invitrogen	12-9852-82	0.25 μg
c-Rel	eFluor660	1RELAH5	Invitrogen	50-6111-80	0.06 μg
pRelB	PE	RelBS552-A7	Invitrogen	MA5-37031	5 μL
GFP	PerCP-eFluor710	5F12.4	Invitrogen	46-6498-80	0.125 μg
Ghost Dye	Violet510		Tonbo	13-0870-T100	1/400
CCR7	PerCP/Cyanine5.5	4B12	Invitrogen	45-1971-82	0.5 μg
T-bet	PE	eBio4B10	Invitrogen	12-5825-82	0.5 μg
GATA-3	Alexa Fluor488	TWAJ	Invitrogen	53-9966-41	0.5 μg
IFNα	FITC	RMMA-1	Pbl	22100-3	0.05 μg
Human-CD123	PE	6H6	Invitrogen	12-1239-42	0.25 μg
Human-CD11c	PerCP-Cyanine5.5	3.9	Invitrogen	46-0116-42	1 μg
Human-CD3	PB	UCHT1	Invitrogen	48-0038-82	0.5 μg
Human-CD19	PB	hib19	Invitrogen	48-0199-42	0.5 μg
Human-CD14	PB	63D3	BioLegend	367122	1 μg
Human-HLA-DR	APC	L243	BioLegend	307610	0.125 μg

Supplementary Table 2: Cytokine information

Cytokine	Source	Catalog#	Concentration
GM-CSF	PeproTech	315-03	20 ng/mL
IFNγ	PeproTech	315-05	20 ng/mL
Flt3L	PeproTech	250-31L	100 ng/mL
SCF	PeproTech	250-03	20 ng/mL
M-SCF	PeproTech	315-02	20 ng/mL
TNF	PeproTech	315-01A	20 ng/mL
IL-1α	PeproTech	211-11A	20 ng/mL
IL-1β	PeproTech	211-11B	20 ng/mL
IL-2	PeproTech	212-12	20 ng/mL
IL-3	PeproTech	213-13	20 ng/mL
IL-4	PeproTech	214-14	5 ng/mL
IL-6	PeproTech	216-16	20 ng/mL
IL-7	PeproTech	217-17	20 ng/mL
IL-9	PeproTech	219-19	50 ng/mL
IL-10	PeproTech	210-10	50 ng/mL
IL-12p70	PeproTech	210-12	50 ng/mL
IL-13	PeproTech	210-13	50 ng/mL
IL-23	Invitrogen	14-8231-63	50 ng/mL
IFNα2	Novoprotein	CK83	50 ng/mL
TGF-β1	Novoprotein	C16W	50 ng/mL
IL-17A	PeproTech	210-17	20 ng/mL

Supplementary Table 3: Primer sequences

**Gene**	**Forward 5'-3'**	**Reverse 5'-3'**
Mouse-*Ehf*	GACCACCCTGTAAAGTCCCA	GAACCTGAAGATGCCTTCCGA
Human-EHF	ACCAAGTACCAGGTGTGGGA	CGCCGTTGATGTCGAACTCT
Mouse-*β-Actin*	GTGACGTTGACATCCGTAAAGA	GCCGGACTCATCGTACTCC
Human-β-Actin	GAAGAGCTACGAGCTGCCTGA	TGATCTTCATTCTGCTGGGTG
VSV	TGATACAGTACAATTATTTTGGGAC	GAGACTTTCTGTTACGGGATCTGG
Mouse-*Ccr7*	TGCACAGACCTGCTGGAAAT	ACGCAGGGACCTATGCAAAT
Mouse-*Cd274*	GTGTGGGCTTTAGCCTTCCT	TTGCATGGGTTGCAGGAGAA
Mouse-*Cd80*	GACAGCGGTGTGTAAGCTGT	ACTAGCTGTGGTCAGGTGGA
Mouse-*Cd86*	AGGGAAACCCCAAAGACAGC	GCTCTCTGGTTGGGAGTGAG
Mouse-*Cd200*	GGAGGCTGACCTCAATGCTA	CATGGAGGACTCAGTAGCCAA
Mouse-*Cd83*	CCGGCGAGTGGAATCTTTGT	GGAGCTAGGAGGTTCCCCTT
Mouse-*Il2ra*	GTATGTGCTTCCTGTCCCCG	TCCTGAGGACTCGCCCTTT
Mouse-*Irf4*	ACTGTTTGTGTCTATGGCATATCAC	CAGACCAATGACTCATTGCAATGAAC
Mouse-*Irf8*	ACACCACAGACGATCCAACC	GTCCTGGAGACATGACAGCC
Mouse-*Rel*	GTGCGCTTACTTCCTCCAGA	GGATTTCTCAGGGACCGACG
Mouse-*Relb*	ACTCACTGAGCCACACACCTCTTC	AGGCTGCTCTTAGGAGTAAGGG
