# Supplementary material for: The transcription factor EHF promotes the maturation and immunosuppression of conventional dendritic cells
Source: Nat Commun. 2026 Feb 23;17:3094. doi: 10.1038/s41467-026-69959-z (PMC13039115; doi:10.1038/s41467-026-69959-z)
Supplement: Supplementary file 2 — Reporting Summary [file 41467_2026_69959_MOESM2_ESM.pdf]

nature portfolio

Corresponding author(s): Cliff Yang

Last updated by author(s): 02/08/2026

Reporting Summary

Nature Portfolio wishes to improve the reproducibility of the work that we publish. This form provides structure for consistency and transparency in reporting. For further information on Nature Portfolio policies, see our Editorial Policies and the Editorial Policy Checklist.

Statistics

For all statistical analyses, confirm that the following items are present in the figure legend, table legend, main text, or Methods section.

n/a	Confirmed	
☐	☒	The exact sample size (n) for each experimental group/condition, given as a discrete number and unit of measurement
☐	☒	A statement on whether measurements were taken from distinct samples or whether the same sample was measured repeatedly
☐	☒	The statistical test(s) used AND whether they are one- or two-sided<br>*Only common tests should be described solely by name; describe more complex techniques in the Methods section.*
☐	☒	A description of all covariates tested
☐	☒	A description of any assumptions or corrections, such as tests of normality and adjustment for multiple comparisons
☐	☒	A full description of the statistical parameters including central tendency (e.g. means) or other basic estimates (e.g. regression coefficient) AND variation (e.g. standard deviation) or associated estimates of uncertainty (e.g. confidence intervals)
☐	☒	For null hypothesis testing, the test statistic (e.g. F, t, r) with confidence intervals, effect sizes, degrees of freedom and P value noted<br>*Give P values as exact values whenever suitable.*
☒	☐	For Bayesian analysis, information on the choice of priors and Markov chain Monte Carlo settings
☒	☐	For hierarchical and complex designs, identification of the appropriate level for tests and full reporting of outcomes
☒	☐	Estimates of effect sizes (e.g. Cohen's d, Pearson's r), indicating how they were calculated

Our web collection on statistics for biologists contains articles on many of the points above.

Software and code

Policy information about availability of computer code

Data collection	FACSDiva v8.0.1, StepOne v2.1. For additional details please refer to Methods.
Data analysis	FlowJo v10.6.2; Prism v10.1.2; Excel v14.3.0; ImageJ 2. For additional details please refer to Methods.

For manuscripts utilizing custom algorithms or software that are central to the research but not yet described in published literature, software must be made available to editors and reviewers. We strongly encourage code deposition in a community repository (e.g. GitHub). See the Nature Portfolio guidelines for submitting code & software for further information.

Data

Policy information about availability of data

All manuscripts must include a data availability statement. This statement should provide the following information, where applicable:

- Accession codes, unique identifiers, or web links for publicly available datasets
- A description of any restrictions on data availability
- For clinical datasets or third party data, please ensure that the statement adheres to our policy

The scRNA-seq data in Fig. 6e (top panel) were downloaded from the Gene Expression Omnibus accession number (GSE94820). The dataset from GSE94820 was analyzed using the R software package Seurat (V4.0), available from CRAN (https://CRAN.R-project.org/package=Seurat).

The scRNA-seq dataset from Fig. 6 and S6, and the CUT&TAG dataset from Figs. 5 and S5 were uploaded to GEO database (GSE260857, GSE260858, GSE260859, GSE260860, GSE260861, GSE275286, GSE275287).

The scRNA-seq data in Fig. 6e (top panel) were previously published,18 and downloaded from the Gene Expression Omnibus accession number (GSE94820). The dataset from GSE94820 was analyzed using the R software package Seurat (V4.0), available from CRAN (https://CRAN.R-project.org/package=Seurat).

The scRNA-seq dataset from Fig. 6 and S6, and the CUT&TAG dataset from Figs. 5 and S5 were uploaded to GEO database (GSE260857, GSE260858, GSE260859, GSE260860, GSE260861, GSE275286, GSE275287).

Research involving human participants, their data, or biological material

Policy information about studies with human participants or human data. See also policy information about sex, gender (identity/presentation), and sexual orientation and race, ethnicity and racism.

Reporting on sex and gender	Not applicable
Reporting on race, ethnicity, or other socially relevant groupings	Not applicable
Population characteristics	Not controlled due to budget constraint
Recruitment	There were no exclusion criteria for healthy volunteers.
Ethics oversight	The human study was approved by the Ethics Committee of the First Affiliated Hospital, Sun Yat-sen University, Guangzhou, Guangdong, China (Approval No. 2022-436). Written informed consent was received prior to participation.

Note that full information on the approval of the study protocol must also be provided in the manuscript.

Field-specific reporting

Please select the one below that is the best fit for your research. If you are not sure, read the appropriate sections before making your selection.

☒ Life sciences ☐ Behavioural & social sciences ☐ Ecological, evolutionary & environmental sciences

For a reference copy of the document with all sections, see nature.com/documents/nr-reporting-summary-flat.pdf

Life sciences study design

All studies must disclose on these points even when the disclosure is negative.

Sample size	No statistical methods were used to predetermine sample size. Sample size were chosen based on the maximum number of mice available at the moment of experiments. Since the mice are on identical genetic backgrounds, a minimum of 3 individual mice were commonly recognized in the field to sufficiently detect differences between genotypes or conditions.
Data exclusions	No data was excluded
Replication	Experiments were replicated successfully for least 3 times, except for sequencing data, as described throughout the paper and in the Methods.
Randomization	Mice of similar ages and sex were used for all the experiments reported. Samples were randomly assigned.
Blinding	For all experiments, the investigators were not blinded to the identities of the samples because treatments and data collection were performed by the same people. During data analysis, investigators were not blinded to group allocation, as this is performed by the same people. The reason for these unblindings is due to a lack of personnel and resources, as investigators have to collect and analysis data by themselves. For human studies, there was no blinding in this study as we did not have different treatment/experimental groups and therefore was not relevant.

Reporting for specific materials, systems and methods

We require information from authors about some types of materials, experimental systems and methods used in many studies. Here, indicate whether each material, system or method listed is relevant to your study. If you are not sure if a list item applies to your research, read the appropriate section before selecting a response.

Materials & experimental systems

n/a	Involved in the study
	[x] Antibodies
	[x] Eukaryotic cell lines
[x]	Palaeontology and archaeology
	[x] Animals and other organisms
[x]	Clinical data
[x]	Dual use research of concern
[x]	Plants

Methods

n/a	Involved in the study
	[x] ChIP-seq
	[x] Flow cytometry
[x]	MRI-based neuroimaging

Antibodies

Antibodies used

The following mouse antibodies for flow cytometry:
CD19 eFluor 450 (Invitrogen, clone: 1D3, Catalog#: 48-0193-82)
CD3ε APC (Invitrogen, clone: 17A2, Catalog#: 17-0032-82)
CD3ε eFluor 450 (Invitrogen, clone: 500A2, Catalog#: 48-0033-82)
F4/80 eFluor 450 (Invitrogen, clone: BM8, Catalog#: 48-4801-82)
TER-119 eFluor 450 (Invitrogen, clone: TER-119, Catalog#: 48-5921-82)
CD11b PerCP-eFluor 710 (Invitrogen , clone: M1/70, Catalog#: 45-0112-82)
CD11b FITC (Invitrogen, clone: M1/70, Catalog#: 11-0112-82)
B220 FITC (Invitrogen, clone: RA3-6B2, Catalog#: 11-0452-82)
CD62L PE-Cyanine7 (Invitrogen, clone: MEL-14, Catalog#: 25-0621-82)
BST-2 FITC (Invitrogen, clone: eBio927, Catalog#: 11-3172-82)
SiglecH APC (Invitrogen, clone: eBio440c, Catalog#: 17-0333-82)
TNF PE-Cyanine7 (Invitrogen, clone: MP6-XT22, Catalog#: 25-7321-82)
Foxp3 PE (Invitrogen, clone: NRRF-30, Catalog#: 12-4771-82)
CD44 APC-Cyanine7 (BioLegend, clone: IM7 Catalog#: 103028)
CD44 APC (BioLegend, clone: IM7, Catalog#: 103012)
B220 PE (BioLegend, clone: RA3-6B2 Catalog#: 103208)
IA/IE APC-Cyanine7 (BioLegend, clone: M5/114.15.2, Catalog#: 107628)
IA/IE PE (BioLegend, clone: M5/114.15.2, Catalog#: 107608)
CD4 PerCP-Cyanine5.5 (BioLegend, clone: GK1.5, Catalog#: 100434)
CD8 BV605 (BioLegend, clone: 53-6.7, Catalog#: 100743)
CD25 FITC (Invitrogen, clone: PC61.5, Catalog#: 11-0251-82)
XCR1 PerCP/Cyanine5.5 (BioLegend, clone: ZET, Catalog#: 148208)
Ly6C FITC (BioLegend, clone: HK1.4, Catalog#: 128006)
Ly6C APC (Invitrogen, clone: HK1.4, Catalog#: 17-5932-82)
F4/80 PE (BioLegend, clone: BM8, Catalog#: 123110)
CD11c APC-Cyanine7 (BioLegend, clone: N418, Catalog#: 117352)
CD45 APC (BioLegend, clone: 30-F11, Catalog#: 103112)
IL12p40 APC (BioLegend, clone: IM7, Catalog#: 505206)
PD-L1 PE-Cy7 (Invitrogen, clone: MIH5, Catalog#: 25-5982-82)
PD-1 APC-Cyanine7(Invitrogen, clone: J43, Catalog#: 47-9985-82)
CD83 PE (Invitrogen, clone: Michel-17, Catalog#: 12-0831-82)
CD80 PE (Invitrogen, clone: 16-10A1, Catalog#: 12-0801-82)
CD86 APC (Invitrogen, clone: GL1, Catalog#: 17-0862-82)
CD200 APC (BioLegend, clone: OX-90, Catalog#: 123810)
CD25 FITC (Invitrogen, clone: PC61.5, Catalog#: 11-0251-82)
CD95 (APO-1/Fas) PE (eBioscience, clone: 15A7, Catalog#: 12-0951-83)
CXCR5 Biotin (BD, clone: 2G8 (RUO), Catalog#: 551960)
GL-7 eFluor 450 (Invitrogen, clone: GL-7, Catalog#: 48-5902-82)
Ly6G/Ly6C eFluor 450 (Invitrogen, clone: RB6-8C5, Catalog#: 48-5931-82)
CD16/CD32 eFluor 450 (Invitrogen, clone: 93, Catalog#: 48-0161-82)
IL-4 APC (Invitrogen, clone: 11B11, Catalog#: 17-7041-82)
IL-2 eFluor 450 (Invitrogen, clone: JES6-5H4, Catalog #: 48-7021-82)
IFN gamma PE (Invitrogen, clone: XMG1.2, Catalog#:12-7311-82)
IL-6 eFluor 450 (Invitrogen, clone: MP5-20F3, Catalog#: 48-7061-82)
IRF4 eFluor 660 (Invitrogen, clone: 3E4, Catalog#: 50-9858-82)
IRF8 PE (Invitrogen, clone: V3GYWCH, Catalog#: 12-9852-82)
c-Rel eFluor 660 (Invitrogen, clone: 1RELAH5, Catalog#: 50-6111-80)
Phospho-RelB (Ser552) PE (Invitrogen, clone: RelBS552-A7, Catalog#: MA5-37031)
GFP PerCP-eFluor710 (Invitrogen, clone: 5F12.4, Catalog#: 46-6498-80)
Ghost Dye Violet 510 (Tonbo Biosciences, Catalog#: 13-0870-T100)
CCR7 PerCP/Cyanine5.5 (Invitrogen, clone: 4B12, Catalog#: 45-1971-82)

T-bet PE (Invitrogen, clone: eBio4B10, Catalog#: 12-5825-82)
GATA-3 Alexa Fluor 488 (Invitrogen, clone: TWAJ, Catalog#: 53-9966-41)
IFNα FITC (Pbl assay science, clone: RMMA-1, #22100-3)

The following human antibodies for flow cytometry:
CD123 PE (Invitrogen, clone: 6H6, Catalog#: 12-1239-42)
CD11c PerCP-Cyanine5.5 (Invitrogen, clone: 3.9, Catalog#: 46-0116-42)
CD3 PB (Invitrogen, clone: UCHT1, Catalog#: 48-0038-82)
CD19 PB (Invitrogen, clone: hib19, Catalog#: 48-0199-42)
CD14 PB (BioLegend, clone: 63D3, Catalog#: 367122,)
HLA-DR APC (BioLegend, clone: L243, Catalog#: 307610)

The following antibodies were used for Western blotting at 1:5000 dilution:
Tubulin (Abcam, host: mouse, #ab78078)
β-actin (Abcam, host: mouse, #ab6276)
Anti-mouse IgG H&L (HRP) (Abcam, host: goat, #ab6789)

For western blotting, mouse monoclonal anti-EHF antibodies were generated in house.

Validation

All antibodies used were commercially available and validated by corresponding manufacturers. The following companies have general validation/reproducibility statements.
BD(https://www.bdbiosciences.com/en-us/products/reagents/flow-cytometry-reagents).
Biolegend (https://www.biolegend.com/en-us/antibodies-reagents/primaries).
Invitrogen (https://www.thermofisher.cn/cn/zh/home/life-science/antibodies.html).
Abcam (https://www.abcam.cn/)

CD19 eFluor 450 (Invitrogen, clone: 1D3, Catalog#: 48-0193-82) https://www.thermofisher.cn/cn/zh/antibody/product/CD19-Antibody-clone-eBio1D3-1D3-Monoclonal/48-0193-82

CD3ε APC (Invitrogen, clone: 17A2, Catalog#: 17-0032-82)

CD3ε eFluor 450 (Invitrogen, clone: 500A2, Catalog#: 48-0033-82) https://www.thermofisher.cn/cn/zh/antibody/product/CD3e-Antibody-clone-eBio500A2-500A2-Monoclonal/48-0033-82

F4/80 eFluor 450 (Invitrogen, clone: BM8, Catalog#: 48-4801-82) https://www.thermofisher.cn/cn/zh/antibody/product/F4-80-Antibody-clone-BM8-Monoclonal/48-4801-82

TER-119 eFluor 450 (Invitrogen, clone: TER-119, Catalog#: 48-5921-82) https://www.thermofisher.cn/cn/zh/antibody/product/TER-119-Antibody-clone-TER-119-Monoclonal/48-5921-82

CD11b PerCP-eFluor 710 (Invitrogen, clone: M1/70, Catalog#: 45-0112-82) https://www.thermofisher.cn/cn/zh/antibody/product/CD11b-Antibody-clone-M1-70-Monoclonal/45-0112-82

CD11b FITC (Invitrogen, clone: M1/70, Catalog#: 11-0112-82) https://www.thermofisher.cn/cn/zh/antibody/product/CD11b-Antibody-clone-M1-70-Monoclonal/11-0112-82

B220 FITC (Invitrogen, clone: RA3-6B2, Catalog#: 11-0452-82) https://www.thermofisher.cn/cn/zh/antibody/product/CD45R-B220-Antibody-clone-RA3-6B2-Monoclonal/11-0452-82

CD62L PE-Cyanine7 (Invitrogen, clone: MEL-14, Catalog#: 25-0621-82) https://www.thermofisher.cn/cn/zh/antibody/product/CD62L-L-Selectin-Antibody-clone-MEL-14-Monoclonal/25-0621-82

BST-2 FITC (Invitrogen, clone: eBio927, Catalog#: 11-3172-82) https://www.thermofisher.cn/cn/zh/antibody/product/CD317-BST2-PDCA-1-Antibody-clone-eBio927-Monoclonal/11-3172-82

SiglecH APC (Invitrogen, clone: eBio440c, Catalog#: 17-0333-82) https://www.thermofisher.com/cn/zh/antibody/product/SIGLEC-H-Antibody-clone-eBio440c-Monoclonal/17-0333-82?imageId=492489

TNF PE-Cyanine7 (Invitrogen, clone: MP6-XT22, Catalog#: 25-7321-82) https://www.thermofisher.com/cn/zh/antibody/product/TNF-alpha-Antibody-clone-MP6-XT22-Monoclonal/25-7321-82?imageId=91487

Foxp3 PE (Invitrogen, clone: NRRF-30, Catalog#: 12-4771-82) https://www.thermofisher.cn/cn/zh/antibody/product/FOXP3-Antibody-clone-NRRF-30-Monoclonal/12-4771-82

CD44 APC-Cyanine7 (BioLegend, clone: IM7 Catalog#: 103028) https://www.biolegend.com/en-us/products/apc-cyanine7-anti-mouse-human-cd44-antibody-3933

CD44 APC (BioLegend, clone: IM7, Catalog#: 103012) https://www.biolegend.com/en-us/products/apc-anti-mouse-human-cd44-antibody-312

B220 PE (BioLegend, clone: RA3-6B2 Catalog#: 103208) https://www.biolegend.com/en-us/products/pe-anti-mouse-human-cd45r-b220-antibody-447

IA/IE APC-Cyanine7 (BioLegend, clone: M5/114.15.2, Catalog#: 107628) https://www.biolegend.com/en-us/products/apc-cyanine7-anti-mouse-i-a-i-e-antibody-5966
IA/IE PE (BioLegend, clone: M5/114.15.2, Catalog#: 107608) https://www.biolegend.com/en-us/products/pe-anti-mouse-i-a-i-e-antibody-367

CD4 PerCP-Cyanine5.5 (BioLegend, clone: GK1.5, Catalog#: 100434) https://www.biolegend.com/en-us/products/percp-cyanine5-5-anti-mouse-cd4-antibody-4220

CD8 BV605 (BioLegend, clone: 53-6.7, Catalog#: 100743) https://www.biolegend.com/en-us/products/brilliant-violet-605-anti-mouse-cd8a-antibody-7636

CD25 FITC (Invitrogen, clone: PC61.5, Catalog#: 11-0251-82) https://www.thermofisher.cn/cn/zh/antibody/product/CD25-Antibody-clone-PC61-5-Monoclonal/11-0251-82

XCR1 PerCP/Cyanine5.5 (BioLegend, clone: ZET, Catalog#: 148208) https://www.biolegend.com/en-us/products/percp-cyanine5-5-anti-mouse-rat-xcr1-antibody-10397

Ly6C FITC (BioLegend, clone: HK1.4, Catalog#: 128006) https://www.biolegend.com/en-us/products/fitc-anti-mouse-ly-6c-antibody-4896

Ly6C APC (Invitrogen, clone: HK1.4, Catalog#: 17-5932-82) https://www.thermofisher.cn/cn/zh/antibody/product/Ly-6C-Antibody-clone-HK1-4-Monoclonal/17-5932-82

F4/80 PE (BioLegend, clone: BM8, Catalog#: 123110) https://www.biolegend.com/en-us/products/pe-anti-mouse-f4-80-antibody-4068

CD11c APC-Cyanine7 (BioLegend, clone: N418, Catalog#: 117352) https://www.biolegend.com/en-us/products/apc-fire-750-anti-mouse-cd11c-antibody-13050

CD45 APC (BioLegend, clone: 30-F11, Catalog#: 103112) https://www.biolegend.com/en-us/products/apc-anti-mouse-cd45-antibody-97

IL12p40 APC (BioLegend, clone: IM7, Catalog#: 505206) https://www.biolegend.com/en-us/products/apc-anti-mouse-il-12-il-23-p40-monomer-dimer-heterodimer-antibody-926

PD-L1 PE-Cy7 (Invitrogen, clone: MIH5, Catalog#: 25-5982-82) https://www.thermofisher.cn/cn/zh/antibody/product/CD274-PD-L1-B7-H1-Antibody-clone-MIH5-Monoclonal/25-5982-82

PD-1 APC-Cyanine7(Invitrogen, clone: J43, Catalog#: 47-9985-82) https://www.thermofisher.cn/cn/zh/antibody/product/CD279-PD-1-Antibody-clone-J43-Monoclonal/47-9985-82

CD83 PE (Invitrogen, clone: Michel-17, Catalog#: 12-0831-82) https://www.thermofisher.cn/cn/zh/antibody/product/CD83-Antibody-clone-Michel-17-Michel17-Monoclonal/12-0831-82

CD80 PE (Invitrogen, clone: 16-10A1, Catalog#: 12-0801-82) https://www.thermofisher.cn/cn/zh/antibody/product/CD80-B7-1-Antibody-clone-16-10A1-Monoclonal/12-0801-82

CD86 APC (Invitrogen, clone: GL1, Catalog#: 17-0862-82) https://www.thermofisher.cn/cn/zh/antibody/product/CD86-B7-2-Antibody-clone-GL1-Monoclonal/17-0862-82

CD200 APC (BioLegend, clone: OX-90, Catalog#: 123810) https://www.biolegend.com/en-us/products/apc-anti-mouse-cd200-ox2-antibody-7338

CD25 FITC (Invitrogen, clone: PC61.5, Catalog#: 11-0251-82) https://www.thermofisher.cn/cn/zh/antibody/product/CD25-Antibody-clone-PC61-5-Monoclonal/11-0251-82

CD95 (APO-1/Fas) PE (eBioscience, clone: 15A7, Catalog#: 12-0951-83) https://www.thermofisher.cn/cn/zh/antibody/product/CD95-APO-1-Fas-Antibody-clone-15A7-Monoclonal/12-0951-83

CXCR5 Biotin (BD, clone: 2G8 (RUO), Catalog#: 551960) https://www.bdbiosciences.com/en-us/products/reagents/flow-cytometry-reagents/research-reagents/single-color-antibodies-ruo/biotin-rat-anti-mouse-cd185-cxcr5.551960?tab=product_details

GL-7 eFluor 450 (Invitrogen, clone: GL-7, Catalog#: 48-5902-82) https://www.thermofisher.cn/cn/zh/antibody/product/GL7-Antibody-clone-GL-7-GL7-Monoclonal/48-5902-82

Ly6G/Ly6C eFluor 450 (Invitrogen, clone: RB6-8C5, Catalog#: 48-5931-82) https://www.thermofisher.cn/cn/zh/antibody/product/Ly-6G-Ly-6C-Antibody-clone-RB6-8C5-Monoclonal/48-5931-82

CD16/CD32 eFluor 450 (Invitrogen, clone: 93, Catalog#: 48-0161-82) https://www.thermofisher.cn/cn/zh/antibody/product/CD16-CD32-Antibody-clone-93-Monoclonal/48-0161-82

IL-4 APC (Invitrogen, clone: 11B11, Catalog#: 17-7041-82) https://www.thermofisher.cn/cn/zh/antibody/product/IL-4-Antibody-clone-11B11-Monoclonal/17-7041-82

IL-2 eFluor 450 (Invitrogen, clone: JES6-5H4, Catalog#: 48-7021-82) https://www.thermofisher.cn/cn/zh/antibody/product/IL-2-Antibody-clone-JES6-5H4-Monoclonal/48-7021-82

IFN gamma PE (Invitrogen, clone: XMG1.2, Catalog#:12-7311-82) https://www.thermofisher.cn/cn/zh/antibody/product/IFN-gamma-Antibody-clone-XMG1-2-Monoclonal/12-7311-82

IL-6 eFluor 450 (Invitrogen, clone: MP5-20F3, Catalog#: 48-7061-82) https://www.thermofisher.cn/cn/zh/antibody/product/IL-6-Antibody-clone-MP5-20F3-Monoclonal/48-7061-82

IRF4 eFluor 660 (Invitrogen, clone: 3E4, Catalog#: 50-9858-82) https://www.thermofisher.cn/cn/zh/antibody/product/IRF4-Antibody-clone-3E4-Monoclonal/50-9858-82

IRF8 PE (Invitrogen, clone: V3GYWCH, Catalog#: 12-9852-82) https://www.thermofisher.cn/cn/zh/antibody/product/IRF8-Antibody-clone-V3GYWCH-Monoclonal/12-9852-82

c-Rel eFluor 660 (Invitrogen, clone: 1RELAH5, Catalog#:50-6111-80) https://www.thermofisher.cn/cn/zh/antibody/product/c-Rel-Antibody-clone-1RELAH5-Monoclonal/50-6111-80

Phospho-RelB (Ser552) (Invitrogen, clone: RelBS552-A7, Catalog#: MA5-37031) https://www.thermofisher.cn/cn/zh/antibody/product/Phospho-RelB-Ser552-Antibody-clone-RelBS552-A7-Recombinant-Monoclonal/MA5-37031
GFP PerCP-eFluor710 (Invitrogen, clone: 5F12.4, Catalog#: 46-6498-80) https://www.thermofisher.cn/cn/zh/antibody/product/GFP-Antibody-clone-5F12-4-Monoclonal/46-6498-80

Ghost Dye Violet 510 (Tonbo Biosciences, Catalog#: 13-0870-T100) https://cytekbio.com/products/ghost-dye-violet-510?variant=40581220302884

CCR7 PerCP/Cyanine5.5 (Invitrogen, clone: 4B12, Catalog#: 45-1971-82) https://www.thermofisher.cn/cn/zh/antibody/product/CD197-CCR7-Antibody-clone-4B12-Monoclonal/45-1971-82

T-bet PE (Invitrogen, clone: eBio4B10, Catalog#: 12-5825-82) https://www.thermofisher.cn/cn/zh/antibody/product/T-bet-Antibody-clone-eBio4B10-4B10-Monoclonal/12-5825-82

GATA-3 Alexa Fluor 488 (Invitrogen, clone: TWAJ, Catalog#: 53-9966-41) https://www.thermofisher.cn/cn/zh/antibody/product/Gata-3-Antibody-clone-TWAJ-Monoclonal/53-9966-41

IFNα FITC (Pbl assay science, clone: RMMA-1, #22100-3) https://www.pblassaysci.com/antibodies/fitc-conjugated-anti-mouse-ifn-alpha-antibody-clone-rmma-1-mab-221003

The following human antibodies for flow cytometry:
CD123 PE (Invitrogen, clone: 6H6, Catalog#: 12-1239-42) https://www.thermofisher.cn/cn/zh/antibody/product/CD123-Antibody-clone-6H6-Monoclonal/12-1239-42

CD11c PerCP-Cyanine5.5 (Invitrogen, clone: 3.9, Catalog#: 46-0116-42) https://www.thermofisher.cn/cn/zh/antibody/product/CD11c-Antibody-clone-3-9-Monoclonal/46-0116-42

CD3 PB (Invitrogen, clone: UCHT1, Catalog#: 48-0038-82) https://www.thermofisher.cn/cn/zh/antibody/product/CD3-Antibody-clone-UCHT1-Monoclonal/48-0038-82

CD19 PB (Invitrogen, clone: hib19, Catalog#: 48-0199-42) https://www.thermofisher.cn/cn/zh/antibody/product/CD19-Antibody-clone-HIB19-Monoclonal/48-0199-42

CD14 PB (BioLegend, clone: 63D3, Catalog#: 367122) https://www.biolegend.com/en-us/products/pacific-blue-anti-human-cd14-antibody-14692
HLA-DR APC (BioLegend, clone: L243, Catalog#: 307610) https://www.biolegend.com/en-us/products/apc-anti-human-hla-dr-antibody-787

The following antibodies were used for Western blotting at 1:5000 dilution:
Tubulin (Abcam, host: mouse, #ab78078) https://www.abcam.cn/products/primary-antibodies/beta-iii-tubulin-antibody-2g10-neuronal-marker-ab78078

β-actin (Abcam, host: mouse, #ab6276) https://www.abcam.cn/products/primary-antibodies/beta-actin-antibody-ac-15-loading-control-ab6276

Anti-mouse IgG H&L (HRP) (Abcam, host: goat, #ab6789) https://www.abcam.cn/products/secondary-antibodies/goat-mouse-igg-h-l-

hrp-ab6789

Anti-EHF antibodies are generated in-house and validation data is provided in Supplementary Figure 5.

Eukaryotic cell lines

Policy information about cell lines and Sex and Gender in Research

Cell line source(s)	293T were purchased from ATCC (CRL-3216), Flt3L-B16 cell line is generated from ATCC-purchased B16 (ATCC, CRL-6475).L929 is purchase from ATCC (CRL-6364).
Authentication	Authentication of 293T ,B16, L929 is provided by ATCC with STR profiling. The complete authentication information is provided at: 293T: https://www.atcc.org/products/all/CRL-6475.aspx; B16: https://www.atcc.org/products/all/CRL-3216.aspx. and L929: https://www.atcc.org/products/all/CRL-6364.aspx.
Mycoplasma contamination	The cell line was confirmed to be negative for mycoplasma contamination by PCR.
Commonly misidentified lines (See ICLAC register)	No commonly misidentified cell lines were used.

Animals and other research organisms

Policy information about studies involving animals; ARRIVE guidelines recommended for reporting animal research, and Sex and Gender in Research

Laboratory animals	All mice were bred and maintained under specific pathogen-free conditions at Shanghai Model Organisms Center Inc., and BSL3 facilities at Sun Yat-sen University, according to the institutional guidelines and protocols approved by the Animal Ethics Committee of Sun Yat-sen University, Guangzhou, Guangdong, China. C57BL/6J (JAX:000664, B6 or WT), B6.Cg-Tg (TcraTcrb) 425Cbn/J (JAX:004194, OT-II), B6.SJL-PtprcaPepcb/BoyJ (JAX:002014, CD45.1+), B6.Cg6-Tg (TcraTcrb) 1100Mjb/J (JAX:003831, OT-I), and B6.Cg-Tg (Itgax-Cre) 1-1Reiz/J (JAX:008068, Itgax-Cre) mice were purchased from the Jackson Laboratory. C57BL/6Smoc-Myd88 em1Smoc (#NM-KO-190192, Myd88 -/-) mice were purchased from Shanghai Model Organisms Center Inc. Age- and sex-matched mice aged 6-16 weeks were used in this study.
Wild animals	This study did not involve the use of wild animals.
Reporting on sex	Sex was not considered during the study; Both sexes were used equally; Due to budgetary concerns, all available mice are used in the study regardless of sex.
Field-collected samples	The study did not involve samples collected from the field.
Ethics oversight	All mice protocols were followed according to the institutional guidelines and protocols approved by the Animal Ethics Committee of Sun Yat-sen University.

Note that full information on the approval of the study protocol must also be provided in the manuscript.

Plants

Seed stocks	No plants were used in the study
Novel plant genotypes	No plants were used in the study
Authentication	No plants were used in the study

ChIP-seq

Data deposition

- [x] Confirm that both raw and final processed data have been deposited in a public database such as GEO.
- [x] Confirm that you have deposited or provided access to graph files (e.g. BED files) for the called peaks.

Data access links *May remain private before publication.*	Lastly, all datasets in this manuscript were deposited at the GEO dataset online (GSE260857, GSE260858, GSE260859, GSE260860, GSE260861, GSE275286, GSE275287):1) https://www.ncbi.nlm.nih.gov/geo/query/acc.cgi?acc=GSE260857<br>2) https://www.ncbi.nlm.nih.gov/geo/query/acc.cgi?acc=GSE260858<br>3) https://www.ncbi.nlm.nih.gov/geo/query/acc.cgi?acc=GSE260859

4) https://www.ncbi.nlm.nih.gov/geo/query/acc.cgi?acc=GSE260860
5) https://www.ncbi.nlm.nih.gov/geo/query/acc.cgi?acc=GSE260861
6) https://www.ncbi.nlm.nih.gov/geo/query/acc.cgi?acc=GSE275286
7) https://www.ncbi.nlm.nih.gov/geo/query/acc.cgi?acc=GSE275287

Files in database submission: all TSV TSV MTX were submitted

Genome browser session (e.g. UCSC): No longer applicable

Methodology

Replicates: No replicates were used .

Sequencing depth: Total effective sequencing base pairs (filtered read length × total number of filtered reads) divided by the total length of the target chromatin region, with the unit "×" (indicating the average number of times each base in the target region is sequenced). Total number reads: IgG (52424470), Ehf fl/fl (75036238), Ehf △CD11C (73251160); uniquely mapped reads: IgG (38156354), Ehf fl/fl (74434474), Ehf △CD11C (72589550).Paired-end Illumina sequencing (Length of reads: 150 bp) using Illumins HiSeq 3000 was performed on the barcoded libraries following the manufacturer's instructions.

Antibodies: The ant-EHF antibodies are generated in house.

Peak calling parameters: For peak calling, the parameters used were macs2 callpeak--shift 0 --extsize 200 --nomodel -B --SPMR -g mm -p 0.05. Peak assignment was performed on the intersected bed file using deepTools: computeMatrix reference-point -p 15 --referencePoint TSS -b 3000 -a 3000 -R ucsc_refseq.bed -S Ehf fl/fl.bw EhfΔCD11C.bw IgG.bw --skipZeros -out ./wt_cko.TSS.gz --outFileSortedRegions ./wt_cko.genes.bed.

Data quality: The raw FASTQ data were first processed through fastp version 0.23.2 with the options: -q 20 -u 50 -n 15 -l 50 and all the downstream analyses were based on high-quality clean data. An index of the reference genome was built and paired-end clean reads were aligned to the reference genome (mm10) using Bowtie2 version 2.2.5 with default parameters. For peak calling, the parameters used were macs2 callpeak--shift 0 --extsize 200 --nomodel -B --SPMR -g mm -p 0.05. Peak numbers: Ehf fl/fl (26633), Ehf △CD11C (1319).

Software: For identification of motifs enriched in peak regions over the background, HOMER's motif analysis (findMotifsGenome.pl), which included known default motifs and de novo motifs, was used with default parameters. The UCSC genome browser was used to visualize the CUT&TAG tracks.

Flow Cytometry

Plots

Confirm that:

- [x] The axis labels state the marker and fluorochrome used (e.g. CD4-FITC).
- [x] The axis scales are clearly visible. Include numbers along axes only for bottom left plot of group (a 'group' is an analysis of identical markers).
- [x] All plots are contour plots with outliers or pseudocolor plots.
- [x] A numerical value for number of cells or percentage (with statistics) is provided.

Methodology

Sample preparation: Cells were blocked with Fc blocker(CD16/32), and stained for specific surface markers. For intracellular staining, cells were fixed and permeabilized and stained for intracellular cytokines by fixation/permeabilization kit (eBiosciences).

Instrument: Flow cytometry data were collected by CytoFLEX (Beckman Coulter) and FACSAria II (BD Biosciences) for cell sorting.

Software: BD FACSDiva software (BD Biosciences) for data acquisition and analyzed using FlowJo software version 10.6.2. Data was graphed using Prism v10.1.2 (Graphpad).

Cell population abundance: After sorting we purity was determined to be >95%.

Gating strategy

The gating strategy is shown in Supplementary figure 8. All antibodies were titrated on murine splenic cells to determine optional concentrations for the separation of positive or negative populations. All population markers were on bifurcated markers and clearly defined positive from negative.

All immune cells are gated with FSC-A/SSC-A for a live gate. Then a viability dye (FVD506) is used to exclude dead cells. Next, FSC-A/FSC-H is used to exclude doublets. After these gating, cells are specifically gated accordingly in Supplementary Information, Gating strategies.

☒ Tick this box to confirm that a figure exemplifying the gating strategy is provided in the Supplementary Information.
